# Preparation of UiO-66-NH_2_@PDA under Water System for Chemical Warfare Agents Degradation

**DOI:** 10.3390/ma14092419

**Published:** 2021-05-06

**Authors:** Mingfei Chen, Yingxue Tu, Songhai Wu

**Affiliations:** 1 School of Chemical Engineering and Technology, Tianjin University, Tianjin 300350, China; 2018207355@tju.edu.cn; 2 Key Laboratory of Functional Polymer Materials of Ministry of Education, Institute of Polymer Chemistry, College of Chemistry, Nankai University, Tianjin 300071, China; tyxdlut@163.com

**Keywords:** metal–organic frameworks, chemical warfare agents, catalytic hydrolysis, polymers

## Abstract

There is an urgent need to develop catalytic degradation technologies for chemical warfare agents (CWAs) that are environmentally friendly and do not require secondary treatment. UiO-66-NH_2_ and other metal–organic frameworks (MOFs) based on zirconium have been shown to promote the catalytic degradation of CWAs. At the same time, MOFs have been studied, and they have shown interesting properties in CWA removal because of their ultrahigh surface area, tunable structures, and periodically distributed abundant catalytic sites. However, MOFs synthesized by conventional methods are mostly powdery crystals that are difficult to process and have poor mechanical stability, which largely limit the development of MOFs in practical applications. An emerging trend in MOF research is hybridization with flexible materials. Polymers possess a variety of unique attributes, such as flexibility, thermal and chemical stability, and process ability, and these properties can be combined with MOFs to make a low-cost and versatile material that also provides convenience for the subsequent integration of such MOFs into independent substrates or textiles. In this article, we used a green and simple method to coat the surface of UiO-66-NH_2_ with polydopamine (PDA), PDA can promote the catalytic hydrolysis of UiO-66-NH_2_ to DMNP (a simulant of chemical warfare agents). Additionally, it can adsorb the toxic hydrolysis product p-nitrophenol, avoiding the trouble of secondary treatment. The half-life of UiO-66-NH_2_ coated with polydopamine (UiO-66-NH_2_@PDA) for catalytic hydrolysis is 8.9 min, and that of pure UiO-66-NH_2_ is 20 min. We speculate that the surface coated with PDA can improve the diffusion of DMNP to the active sites of UiO-66-NH_2_.

## 1. Introduction

CWAs are some of the deadliest toxins in the world, e.g., organophosphates [1,2,3,4,5]. The organophosphates currently used as chemical warfare agents have the ability to quickly inhibit acetylcholinesterase, causing the excessive accumulation of acetylcholine in the body. This causes serious disorders of the central and peripheral cholinergic nervous system, and it eventually leads to death [3]. The destruction of these CWAs and other toxins is an important societal challenge. Currently available CWA degradation techniques include incineration, water hydrolysis followed by biotreatment, and water hydrolysis followed by super critical water oxidation. Drawbacks to these techniques include the selectivity to the analyte, the degradation of the enzyme over extended treatment time, and the lack of robustness for practical applications.

Nanostructured materials have proven to be suitable for the degradation of a variety of pollutants via heterogeneous catalysis and may be viable for the degradation of CWAs [6]. MOFs, compounds assembled from the coordination of organic linkers and metal-containing secondary building units [7,8,9], have been studied and have shown interesting properties in CWA removal because of their ultrahigh surface area, tunable structures, and periodically distributed abundant catalytic sites [10]. In particular, the zirconium-based MOF UiO-66-NH_2_ [11] has been shown to have good reactivity with CWAs. However, MOFs synthesized by conventional methods are mostly powdery crystals that are difficult to process and have poor mechanical stability, thus largely limiting the development of MOFs in practical applications. An emerging trend in MOF research is hybridization with flexible materials. As polymers possess a variety of unique attributes [12], such as flexibility, thermal and chemical stability, and process ability. Previous work has shown that assembling a polymer and an MOF is an effective method because a polymer composite can actually enhance the characteristics of MOFs through framework stabilization [13] or the enhanced uptake of a desired analyte [11]. Significant advances have been made in the field of MOF composites by achieving the covalent integration of MOF and polymer components.

Dopamine (DA), a neurotransmitter, widely exists in the animal brain [14] and belongs to a class of catecholamines with excellent biocompatibility. It has drawn great attention since it contains plenty of amine and catechol functional groups and has the ability to adhere to the surface of many materials and form adhesive coatings on a wide range of substrates [15,16,17]. Notably, it can be self-polymerized to form PDA under mild conditions (weakly alkaline pH) [18,19]. This process is simple, green, and low-cost. The abundant functional groups, especially catechol groups [20], of PDA are expected to be the active sites for heavy metals ions, synthetic dyes, and other organic pollutants through electrostatic, bidentate chelating, or hydrogen bonding interactions [21,22].

In this work, we used a green and simple method to coat PDA on the surface of UiO-66-NH_2_ to form a composite material of UiO-66-NH_2_@PDA. We took a typical type of CWA, the organophosphate compound methyl paraoxon (DMNP), as a simulant and further explored the effect of the UiO-66-NH_2_@PDA composite material on the catalytic degradation of DMNP. Studies have shown that the catalytic degradation of the UiO-66-NH_2_@PDA composite material has a half-life of 8.9 min, and the catalytic degradation of UiO-66-NH_2_ has a half-life of 20 min. In addition, PDA coated on the surface of UiO-66-NH_2_ can adsorb p-nitrophenol, the toxic degradation product of DMNP, thus avoiding the trouble of secondary treatment.

## 2. Experimental Section

### 2.1. Chemicals and Materials

2-aminoterephthalic acid (H_2_BDC-NH_2_), dopamine hydrochloride, and zirconium(IV) chloride (ZrCl_4_) were purchased from Beijing Bailingwei, Beijing, China. Glacial acetic acid (CH_3_COOH), dilute hydrochloric acid(HCl), and hydroxymethyl aminomethane (Tris) were purchased from Shanghai Aladdin Biotechnology Co., Ltd, Shanghai, China. Methanol and N,N-dimethylformamide (DMF) were purchased from Tianjin Concord Technology Co., Ltd., Tianjin, China, and N-ethylmorpholine and dimethyl 4-nitrophenylphosphate (methyl paraoxon, DMNP) were purchased from Shanghai Macklin Biochemical Co., Ltd., Shanghai, China. All raw materials were used without further purification.

### 2.2. Synthesis of UiO-66-NH_2_

UiO-66-NH_2_ was solvent thermal synthesized based on the method of Dr. Guang Lu et al. [23]. Zirconium chloride (ZrCl_4_; 279.4 g) and 2-aminoterephthalic acid (H_2_BDC-NH_2_; 217.4 mg) were dissolved in a mixed solution of N,N-dimethylformamide (DMF; 140 mL) and glacial acetic acid (CH_3_COOH; 0.36 mol). Then, the mixture was transferred into a 250 mL Teflon-lined stainless-steel autoclave for a homogeneous reaction that was maintained at 393 K for 24 h. It was cooled to ambient temperature and repeatedly washed three times with DMF and methanol. A white crystal was obtained by drying at 353 K.

### 2.3. Preparation of Buffer Solution

Hydroxymethyl aminomethane (Tris, 1.211 g) were dissolved in ultra-pure water (200 mL). Then, the mixture was transferred into a 1 L volumetric flask, the volume was made with ultra-pure water, and the pH was adjusted to 8.5 with dilute hydrochloric acid (HCl).

### 2.4. Synthesis of UiO-66-NH_2_@PDA Composites

Dopamine hydrochloride (DA, 100 mg) was dissolved in the above-mentioned buffer solution (200 mL, dilution can get different DA concentration solutions) and stirred for 1 min, the solution turned brown. Additionally, then UiO-66-NH_2_ (50 mg) were dispersed in the mixture and subjected to ultrasound for 10 min; afterwards, the solution turned white-brown and was stirred for 24 h at the room temperature. After the reaction, we centrifuged the resulting solution to obtain a black solid and washed it three times with ultra-pure water. The black solid was obtained by drying at 353 K.

### 2.5. Characterizations

The morphology and microstructure of the samples were characterized using a field emission scanning electron microscope (JSM-7500F, JEOL, Kyoto, Japan) and a transmission electron microscope (FEI Talos F200X G2, Philips-FEI, Amsterdam, The Netherlands). Energy-dispersive X-ray analysis (EDS) was carried out by the model MIRA III (TESCAN, Shanghai, China). Low-angle X-ray diffraction (XRD) patterns of the samples were detected by X-ray diffractometer (SmartLab, JEOL, Kyoto, Japan). Powder X-ray diffraction (PXRD) measurements were taken using a X-ray powder diffractometer (Miniflex 600, Rigaku, Tokyo, Japan). The FT-IR spectra of samples in the range of the 4000–400 cm^−1^ wave number were obtained on a TENSOR II model FT-IR spectrometer (BRUKER, Berlin, Germany). The X-ray photoelectron spectroscopy (XPS) of the product was shown on applied electron spectrometers (ESCALAB 250XI, Thermo, Waltham, MA, USA). The thermogravimetric (TG-DSC) analysis of nanocomposites was conducted on a TA thermogravimeter (Netzsch, Berlin, Germany) with air from room temperature to 1200 °C and a heating rate of 10 °C/min. UV–visible absorption spectra were obtained using a U-4100 spectrophotometer (Hitachi, Tokyo, Japan).

### 2.6. Degradation of DMNP

DMNP degradation experiments were carried out at room temperature via a method similar to that reported by Katz et al. [24]. Initially, 50 mg of a solid sample were introduced to a 0.45 M aqueous solution of N-ethylmorpholine (100 mL). The mixture was stirred until the solid completely evenly dispersed in the solution. Then, 400 μL of the above-mentioned solution were introduced to a 4 mL vial. After that, 100 μL of DMNP (1 mg·mL^−1^) was also introduced to the 4 mL vial and continuously stirred (1100 rpm) over the course of the experiment. 50 μL aliquots were extracted periodically over 120 min. The aliquots were diluted to 1 mL with 0.45 M aqueous N-ethylmorpholine and measured using UV-vis spectroscopy. The concentration of DMNP was determined by Lambert–Beer’s law based on the absorbance at 266 nm. The percent conversion of DMNP was calculated from the concentration ratio of degraded DMNP to the initial DMNP. Standard control reactions were performed under the same conditions. 

## 3. Results and Discussion

**Figure 1:**
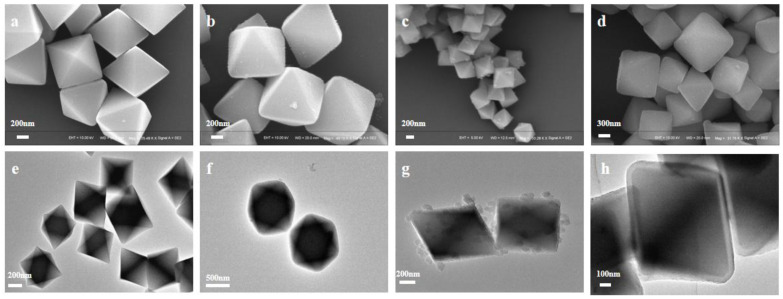
SEM and TEM imaging of (**a**,**e**) UiO-66-NH_2_ and (**b**,**f**) UiO-66-NH_2_@PDA, where the DA concentration is 0.1 mg·mL^−1^; (**c**,**g**) UiO-66-NH_2_@PDA, where the DA concentration is 0.25 mg·mL^−1^; and (**d**,**h**) UiO-66-NH_2_@PDA, where the DA concentration is 0.5 mg·mL^−1.^

UiO-66-NH_2_ and UiO-66-NH_2_@PDA were characterized by SEM and TEM. It could be seen that we successfully prepared octahedron UiO-66-NH_2_ with regular crystal faces and relatively uniform size, with an average particle size of around 600 nm. In order to better coat PDA onto the surface of UiO-66-NH_2_, we explored the coating effect under different DA concentrations using SEM and TEM (Figure 1a–h). When the DA concentration reached 0.25 mg·mL^−1^, obvious small spheres began to appear on the surface of UiO-66-NH_2_, which was presumably formed by the self-aggregation of DA. When the DA concentration reached 0.5 mg·mL^−1^, the polymer pellets on the surface disappeared. According to the SEM and TEM images, it was speculated that the amount of DA was enough to adhere to UiO-66-NH_2_ and form a coat of polymer. By comparing the SEM and TEM images of Figure 1a,b,e,f, we can observe that there was almost no trace of PDA on the surface of UiO-66-NH_2_ at this time. This may have been because the concentration of DA was too low to allow visible polymers to form on the surface of UiO-66-NH_2_.

**Figure 2:**
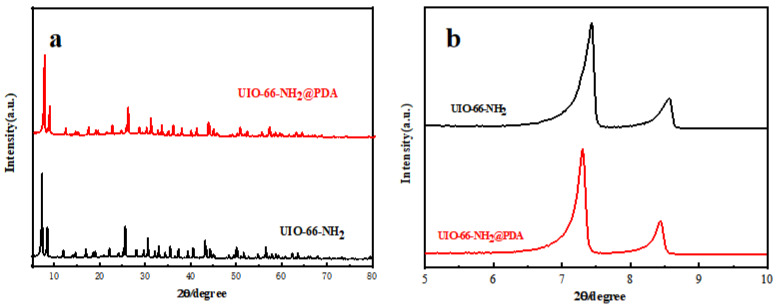
(**a**) XRD patterns of UiO-66-NH_2_ and UiO-66-NH_2_@PDA; (**b**) low-angle XRD patterns of UiO-66-NH_2_ and UiO-66-NH_2_@PDA.

UiO-66-NH_2_ and UiO-66-NH_2_@PDA (all subsequent tests used a 0.5 mg·mL^−1^ DA concentration for UiO-66-NH_2_@PDA) were characterized by XRD to determine the crystalline of the materials. The XRD pattern of the as-synthesized UiO-66-NH_2_ corresponded well to those reported previously [10,25], demonstrating the successful synthesis of UiO-66-NH_2_. Figure 2a,b shows that the diffraction maximum similarity of UiO-66-NH_2_ and UiO-66-NH_2_@PDA was very high, which proved that PDA coating on the surface did not affect the crystal structure of UiO-66-NH_2_.

**Figure 3:**
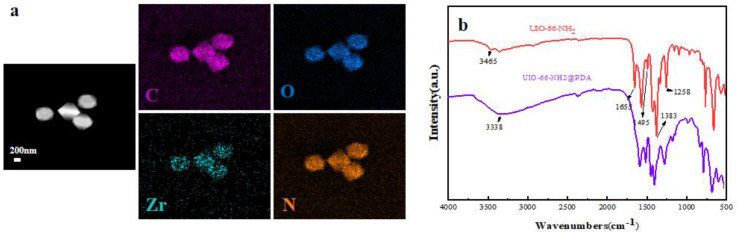
(**a**) EDS element mapping of UiO-66-NH_2_@PDA; (**b**) FT-IR spectra of UiO-66-NH_2_ and UiO-66-NH_2_@PDA.

The EDS elemental mapping of UiO-66-NH_2_@PDA showed that the Zr and O elements were more distributed in the core region, whereas the elements C and N were more distributed in the peripheral region (Figure 3a). FT-IR was applied to further characterize UiO-66-NH_2_ and UiO-66-NH_2_@PDA, as shown in Figure 3b. The characteristic peaks of UiO-66-NH_2_ were identified as follows. The symmetric and asymmetric N-H stretching modes were found at 3360 cm^−1^ and 3465 cm^−1^, respectively [26]. In addition, the bonding between aromatic carbon and nitrogen, C–N, could be also observed at 1258 and 1383 cm^−1^ [27]. The peaks at 1495 cm^−1^ were the result of aromatic C–C ring stretching, and those at 656 cm^−1^ arose from the Zr–μ–Zr stretching inside the node [28]. The broad absorption of the O–H stretching near 3338 cm^−1^ became stronger after the PDA coating. Additionally, the peak of C=O stretching vibrations at 1655 cm^−1^ decreased largely due to the reduction of UiO-66-NH_2_ by PDA, and the peak of C–O–H 1160 cm^−1^ emerged in the FT-IR spectrum of UiO-66-NH_2_@PDA, indicating the presence of UiO-66-NH_2_ coated with PDA [29,30]. In the FT-IR spectra of the UiO-66-NH_2_ and UiO-66-NH_2_@PDA, not only did the peaks representing the groups of C–N, N–H, and C–O–H decrease in intensity but also their location shifted, from which we speculated that PDA was successfully deposited on the surface of the UiO-66-NH_2_ with the aid of the metal-binding ability of catechol and nitrogen-containing groups present in the PDA structure.

**Figure 4:**
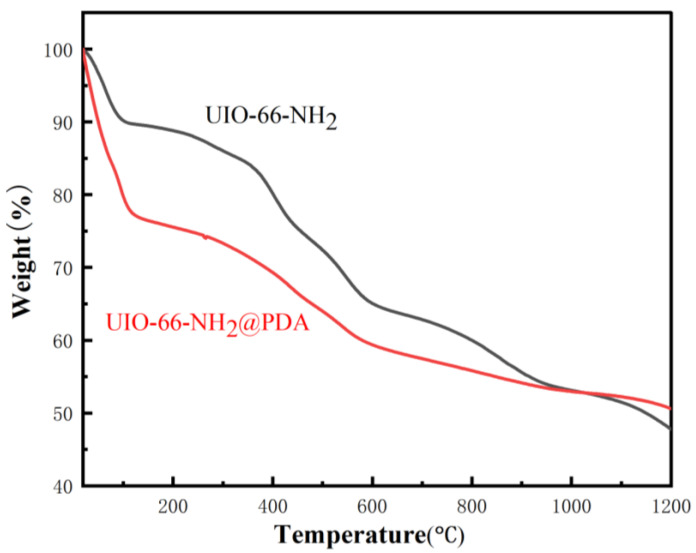
TGA curves of pure UiO-66-NH_2_ and UiO-66-NH_2_@PDA.

Figure 4 shows that between 30 and 120 °C, both UiO-66-NH_2_ and UiO-66-NH_2_@PDA had a similar slight downward trend, which was primarily due to residual organic solvent in the sample’s pores. The weight loss of the sample was caused by the volatilization of a small amount of organic solvent. Between 120 and 600 °C, the weight loss of UiO-66-NH_2_ was primarily due to the collapse of the framework structure, supplemented by the formation of oxides; after 600 °C, the weight loss of UiO-66-NH_2_ was primarily due to the formation of oxides, supplemented by the collapse of the framework structure. When the weight loss of the sample no longer changed, the framework structure of UiO-66-NH_2_ had completely collapsed, and all the samples were zirconium oxides. Compared with UiO-66-NH_2_ and UiO-66-NH_2_@PDA, the thermogravimetric curves indicate that the mass change behavior of UiO-66-NH_2_@PDA coated with PDA varied from that of pure UiO-66-NH_2_. This also demonstrates the presence of PDA on the surface of UiO-66-NH_2_.

**Figure 5:**
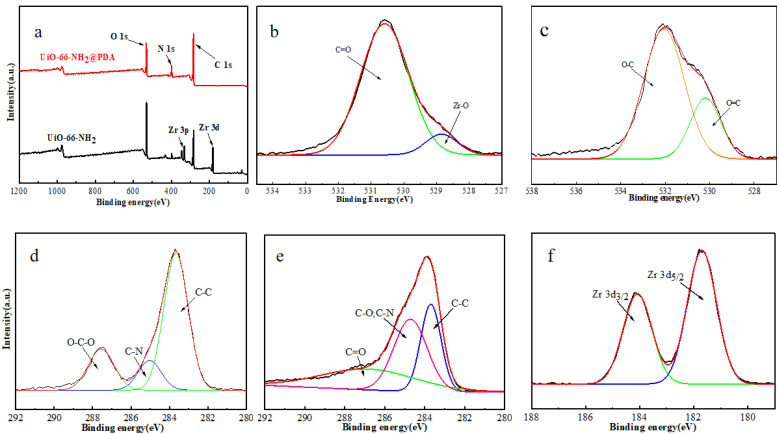
XPS survey spectra of: (**a**) UiO-66-NH_2_ and UiO-66-NH_2_@PDA nanostructures, (**b**) high-resolution O 1s core level for UiO-66-NH_2_, (**c**) high-resolution O 1s core level for UiO-66-NH_2_@PDA, (**d**) high-resolution C 1s core level for UiO-66-NH_2_, (**e**) high-resolution C 1s core level for UiO-66-NH_2_@PDA, and (**f**) high-resolution Zr 3d core level for UiO-66-NH_2_.

As shown in Figure 5a, UiO-66-NH_2_ demonstrated O 1s, N 1s, Zr 3d, and C 1s, while after the deposition of PDA onto UiO-66-NH_2_, the Zr 3d peak disappeared. This also confirmed that the surface of UiO-66-NH_2_ was coated with a certain thickness of PDA [31]. Additionally, the peaks at 529.4 and 530.5 eV were related to the Zr–O and C–O bonds in UiO-66-NH_2_ [25,32], respectively, in Figure 5b. After PDA layer formation onto UiO-66-NH_2_, the deconvolution of O 1s was related to C–O and C=O bonds in Figure 5c. Additionally, the deconvolution of the C 1s region related to UiO-66-NH_2_ and UiO-66-NH_2_@PDA nanostructures was recorded (Figure 5d,e). As seen here, PDA presented characteristic peaks at 283.5, 284.8, and 287.5 eV that corresponded to the C–C, C–O, C–N, and C=O bonds (Figure 3e) [33,34], indicating the successful formation of PDA on the surface of UiO-66-NH_2_. Furthermore, UiO-66-NH_2_ demonstrated peaks at 283.5, 284.9, and 287.5 eV, which were attributed to C–C, C–N, and O–C–O, respectively, in which the new O–C–O peak belonged to organic linker carboxylate groups (Figure 5d) [31]. Finally, the presence of Zr 3d3/2 (184.5 eV) and Zr 3d5/2 (181.7 eV) peaks related to the spin orbitals of UiO-66-NH_2_ nanostructures is demonstrated in Figure 5f [35]. The obtained XPS results were in good agreement with the XRD pattern of UiO-66-NH_2_@PDA, which clearly indicated the successful fabrication of targeted samples.

**Figure 6:**
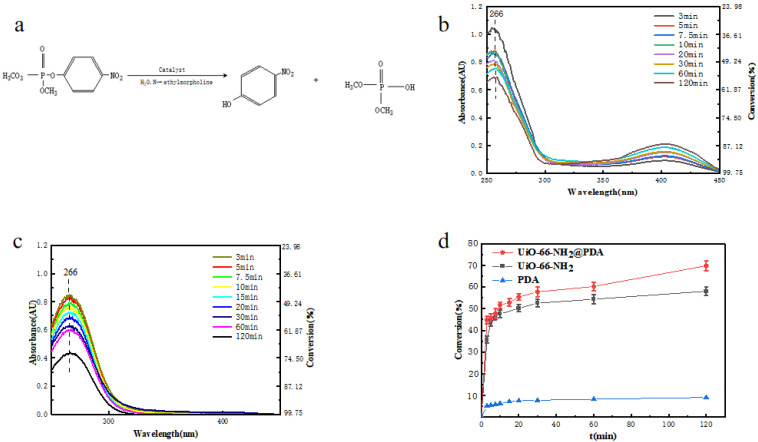
(**a**) Catalytic hydrolysis mechanism of DMNP; (**b**) UV–vis spectra of the degradation of DMNP with UiO-66-NH_2_ at room temperature; (**c**) UV–vis spectra of the degradation of DMNP with UiO-66-NH_2_@PDA at room temperature; (**d**) UiO-66-NH_2_, PDA, and UiO-66-NH_2_@PDA degradation of DMNP conversion rate under the same conditions comparison.

The catalytic hydrolysis mechanism of DMNP is shown in Figure 6a. A common mechanism for the hydrolysis of DMNP is that first, the oxygen in the phosphorus–oxygen bond binds to the unsaturated coordination site on the Lewis acidic metal cation, which is accompanied by the attenuation of the phosphorus–oxygen bond [36]. Then, the phosphate receives a metal-bound or free hydroxide anion. Finally, the catalyst is regenerated by the dissociation of nontoxic products from the active site. The Zr_6_O_4_(OH)_4_ node contains enzyme-like bimetallic Lewis acidic metal centers bridged by a hydroxide, which are effective for the cleavage of P–O bonds, and the amine moiety in UiO-66-NH_2_, as a Brønsted base, can enhance the catalytic activity through the transfer of a proton during the catalytic cycle [36,37,38]. The reaction requires the presence of N-ethylmorpholine as a buffer solution. The buffer acts to remove acidic byproducts from the reaction, as well as to deprotonate water molecules and facilitate the reaction [39].

We also used UiO-66-NH_2_@PDA and pure UiO-66-NH_2_ as examples to compare their application effects in the degradation of DMNP based on the above-mentioned characterization and analysis. During the reaction, the catalyst and DMNP were controlled in the same amount, and the reaction was carried out at room temperature. The catalytic degradation half-life and reaction rate of UiO-66-NH_2_ and UiO-66-NH_2_@PDA were compared. Katz et al. reported that UiO-66 powder exhibited a half-life of 45 min for the degradation of DMNP in 0.45 M N-ethylmorpholine [39]. Figure 6b,c demonstrates how the degradation efficiency of DMNP differed with time under various conditions, as we diluted the reaction solution with N-ethylmorpholine/water buffer solution and then measured its absorption spectrum with a UV–vis spectrophotometer. DMNP has a UV absorption peak of around 266 nm, while p-nitrophenol, a degradation product of DMNP, has a UV absorption peak of around 403 nm. We can see the presence of p-nitrophenol as a hydrolysate in Figure 6b. Since PDA adsorbed it, we cannot see the product’s adsorption peak at 403 nm in Figure 6c, which also confirms that the surface-coated PDA could adsorb p-nitrophenol.

Within the first 3 min of contact between the UiO-66-NH_2_ and DMNP, a significant decrease in the reactant peak at 266 nm was observed, likely due in large part to the adsorption of DMNP. At the same time, the p-nitrophenol peak at 403 nm increased, as seen in Figure 6b. There was considerable complexity to the kinetics of the overall reaction, where it is believed that heterogeneous binding occurs during the adsorption of DMNP [6]. As the reaction progressed, the reaction rate began to decrease by about 20 min and tended to be flat. We speculate that this was due to the decrease of the DMNP concentration of the reactant and the close-to-saturation adsorption of DMNP by the catalyst. In this regard, it seems that the reaction could be promoted by increasing the content of the catalyst. The half-life of UiO-66-NH_2_ degrading DMNP at room temperature was found to be 20 min (the initial concentration of DMNP was 10 mg·L^−1^), according to the kinetic calculation results in Figure 6b,c. The half-life of UiO-66-NH_2_@PDA degrading DMNP was found to be 8.9 min under the same conditions, which was slightly shorter than that of UiO-66-NH_2_, and the degradation performance was faster. Compared with the half-life of the degradation of neurotoxic agent coated with polyethyleneimine on the surface of NU-1000, which was 12.7 min, the half-life was improved [40]. By 120 min, the remaining DMNP concentration in UiO-66-NH_2_@PDA was 3.0 mg·L^−1^ (the conversion rate of DMNP was 70%), while the corresponding UiO-66-NH_2_ remaining DMNP concentration was 4.2 mg·L^−1^ (the conversion rate of DMNP was 58%, ignoring the change in solution volume caused by taking the point). In addition, the performance of UiO-66-NH_2_@PDA was better than UiO-66-NH_2_. This behavior may have been due to the ability of PDA to allow DMNP to better diffuse and approach the active site on UiO-66-NH_2_. In contrast, the PDA sample without UiO-66-NH_2_ exhibited a negligible amount of degradation of DMNP, as seen in Figure 6d, thus indicating these samples are not capable of degrading DMNP without the presence of UiO-66-NH_2_. The resulting solution showed a visible yellow color due to the presence of p-nitrophenol, as seen in Figure 6b.

The UiO-66-NH_2_@PDA used in this experiment had the benefit of being able to efficiently degrade DMNP, and it combined PDA with good flexibility, process ability, and adsorption of DMNP degradation products like p-nitrophenol, which is also toxic, thus avoiding the need for secondary treatment.

## 4. Conclusions

In summary, we successfully synthesized a rationally designed UiO-66-NH_2_@PDA nanoparticle, with PDA coated on the surface, that exhibited an enhanced catalytic efficiency toward CWA degradation under the same conditions. We speculate that the surface-coated PDA enhanced the dispersibility of MOF, allowing the reactants to reach the active site faster. Additionally, the PDA could avoid the secondary treatment of p-nitrophenol and reduce costs. As-synthesized UiO-66-NH_2_@PDA samples were characterized using XRD and FT-IR, thus demonstrating that the structural integrity of the MOF was maintained during the synthesizing process.

## Data Availability

The data presented in this study are available on request from the corresponding author data.
